# Bio-inspired feedback-circuit implementation of discrete, free energy optimizing, winner-take-all computations

**DOI:** 10.1007/s00422-016-0684-8

**Published:** 2016-03-29

**Authors:** Tim Genewein, Daniel A. Braun

**Affiliations:** Max Planck Institute for Biological Cybernetics, Spemannstr. 38, 72076 Tübingen, Germany; Max Planck Institute for Intelligent Systems, Tübingen, Germany; Graduate Training Centre of Neuroscience, Tübingen, Germany

**Keywords:** Analog circuits, Competition, Integration, Bayesian inference, Free energy

## Abstract

Bayesian inference and bounded rational decision-making require the accumulation of evidence or utility, respectively, to transform a prior belief or strategy into a posterior probability distribution over hypotheses or actions. Crucially, this process cannot be simply realized by independent integrators, since the different hypotheses and actions also compete with each other. In continuous time, this competitive integration process can be described by a special case of the replicator equation. Here we investigate simple analog electric circuits that implement the underlying differential equation under the constraint that we only permit a limited set of building blocks that we regard as biologically interpretable, such as capacitors, resistors, voltage-dependent conductances and voltage- or current-controlled current and voltage sources. The appeal of these circuits is that they intrinsically perform normalization without requiring an explicit divisive normalization. However, even in idealized simulations, we find that these circuits are very sensitive to internal noise as they accumulate error over time. We discuss in how far neural circuits could implement these operations that might provide a generic competitive principle underlying both perception and action.

## Introduction

The competition for limited resources is a central theme in biology. In evolutionary theory, the competition for limited resources enforces the process of natural selection, where differential reproductive success of different genotypes lets some genotypes increase their share in the overall population, while others are driven to extinction [5]. This process can be modeled by the replicator equation that quantifies how the proportion of a particular genotype evolves over time depending on the fitness of all other genotypes, such that genotypes achieving more than the average fitness proliferate, and genotypes that perform below average recede [53, 78]. From a mathematical point of view, each genotype can be considered as a hypothesis that accumulates evidence and where different hypotheses compete for probability mass, since the probabilities of all hypotheses must always sum to unity [66].

Also, ontogenetic processes underlying action and perception are governed by competition for limited resources. Well-known examples of competition include binocular rivalry [72], bistable perception [38], attention [20, 22] or affordance competition for action selection [17]. In particular, the process of perception is often understood as an inference process where sensory ambiguity is resolved by competing “hypotheses” that accumulate evidence on the time scale of several hundred milliseconds [37, 42]. A quantitatively well-studied example is the random-dot motion paradigm [11, 29], where subjects observe a cloud of randomly moving dots with a particular degree of motion coherency before they have to decide whether the majority of dots moved to the right or to the left. Depending on the degree of coherency, this evidence accumulation process proceeds faster or slower. Moreover, in this paradigm, neural responses in sensory cortical areas have been shown to be consistent with encoding of log-odds between different hypotheses, thereby reflecting the competitive nature of the evidence accumulation process [29]. Intriguingly, it can be shown that such a process of competitive evidence accumulation is formally equivalent to natural selection as modeled by the replicator dynamics [31, 66].

The problem of acting can be conceptualized in a similar way as the inference process [25, 36, 57, 70, 71, 73, 76]. An actor chooses between different competing actions and wants to select the action that will bring the highest benefit. Even in the absence of any perceptual uncertainty, an actor with limited information processing capabilities might not be able to select the best action—for example, when planning the next move in a chess game—because the number of possibilities exceeds what the decision-maker can consider in a given time frame. Such bounded decision-makers can sample the action space according to some prior strategy during planning and can only realize strategies that do not deviate too much from their prior [56, 59]. If this deviation is measured by the relative entropy between prior and posterior strategy, then the competition between actions is determined by the accumulated utility of each action in the planning process. In this framework, action and perception can be described by the same variational principle that takes the form of a free energy functional [10, 27, 55, 58].

**Fig. 1:**
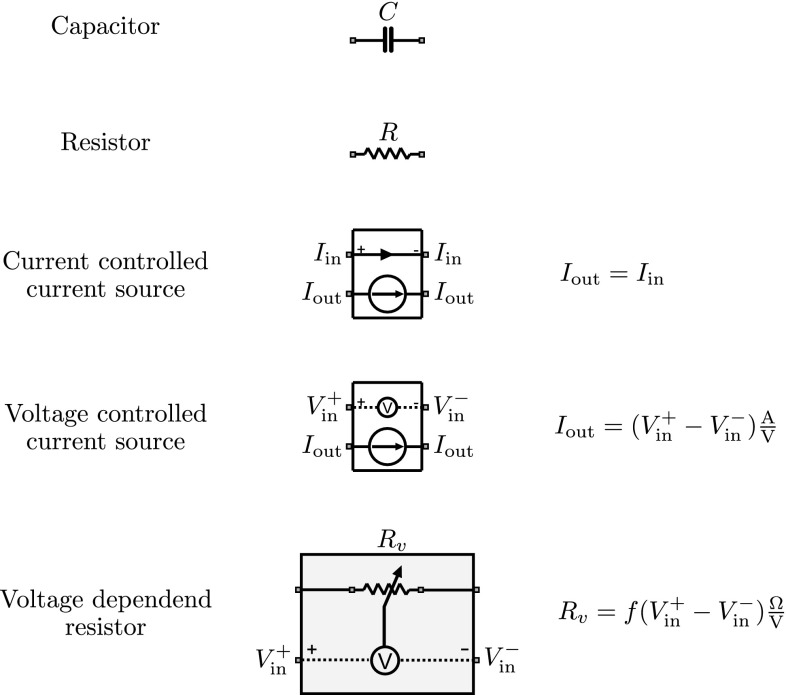
Schematic element representations. The set of biologically interpretable building blocks includes capacitors, resistors, controlled current sources and voltage-dependent conductances. The *circled* *V* indicates a voltmeter. *Arrows* on currents indicate polarity. The function *f* in the variable conductance can represent different mappings depending on the context

In this study, we investigate how such competitive accumulation processes could be physically implemented. In particular, we are interested in the design of bio-inspired analog electric circuits that are made of components that are interpretable in relation to possible neural circuits. The components one typically finds in equivalent circuit diagrams of single neurons in textbooks are capacitors, resistors, voltage-dependent conductances and voltage sources [19, 51]. In order to allow for relay of currents between different neurons, we also allow for copy elements implemented by voltage- or current-controlled current sources that have fixed input-output relationships. In the following, we are interested in bio-inspired analog circuit designs that implement free energy optimizing dynamics, but whose components are restricted to this biologically motivated set of building blocks Fig 1. From a biological point of view, the appeal of such circuits is that they intrinsically perform normalization and do not require an explicit computational step for divisive normalization [14]. In particular, we assume in the following that there are a finite number of incoming input streams that are represented by time-dependent physical signals. These signals are accumulated competitively over time by a finite number of integrators that represent a free energy optimizing posterior distribution over the integrated inputs. The aim of the paper is to investigate the biological plausibility of circuit designs for such competitive evidence accumulation where integration and competition are implemented in the same process without the need for a separate process for explicit normalization.

## Results

### The frequency-independent replicator equation

Both Bayesian inference [7] and decision-making with limited information processing resources [58] may be written as an update equation of the following form

1 $$\begin{aligned} p_{t+1}(x) = p_t(x) \exp \left( \alpha \left( \Delta W_t(x) - \Delta F_t \right) \right) \end{aligned}$$

where $\Delta F_t = \frac{1}{\alpha } \log \sum _x p_t(x) \exp (\alpha \Delta W_t(x) )$ is required for normalization and $\alpha$ is a temperature parameter. Equation (1) describes the update from a prior $p_t(x)$ to a posterior $p_{t+1}(x)$. This update can also be formalized as a variational principle in the posterior probability, where

2 $$\begin{aligned} p_{t+1}(x)= & {} \arg \max _{q(x)}\nonumber \\&\times \left\{ \sum _x q(x) \Delta W_t(x) - \frac{1}{\alpha } \sum _x q(x) \log \frac{q(x)}{p_t(x)} \right\} \nonumber \\ \end{aligned}$$

extremizes a free energy functional. In the case of inference, the distribution $p_t(x)$ indicates the prior probability of hypothesis *x* at time *t* and $\Delta W_t(x)= \log p(D|x)$ represents new evidence that comes in at time *t* given by the log-likelihood $\log p(D|x)$. In this case, the optimization implicit in Eq. (2) underlies approximate Bayesian inference, and in particular variational Bayes methods. In the case of acting, the distribution $p_t(x)$ indicates a prior strategy of sampling actions *x* and $\Delta W_t(x)$ represents the utility gain of choosing action *x*. In either case, the total utility or total evidence of *x* changes from a previous state of absolute utility or evidence $W_t(x)$ to a new value of $W_{t+1}(x)=W_t(x)+\Delta W_t(x)$. The subtraction of the free energy difference $\Delta F_t$ leads to competition between the different hypotheses or actions *x*, if $\Delta W_t(x) > \Delta F_t$ the hypothesis or action *x* gains probability mass, if $\Delta W_t(x) < \Delta F_t$ it loses probability mass; in the case of $\Delta W_t(x) = \Delta F_t$, the probability mass remains constant.

One of the problems with Eq. (1) is that it is not straightforward how to implement the computation of the normalization $\Delta F_t$. However, in continuous time this computation simplifies to computing an expectation value. In the limit of infinitesimally small time steps in Eq. (1), one arrives at the continuous update equation

3 $$\begin{aligned} \frac{\partial p(x,t)}{\partial t} = \alpha \,\, p(x,t) \left( \frac{\partial W(x,t)}{\partial t} - \sum _{x^{\prime }} p(x^{\prime },t) \frac{\partial W(x^{\prime },t)}{\partial t} \right) \! . \end{aligned}$$

Equation (3) is a special case of the replicator equation used in evolutionary game theory to model population dynamics. In evolutionary game theory, the probability *p*(*x*, *t*) indicates the frequency of type *x* in the total population at time *t* and ${\partial W(x,t)}/{\partial t}$ corresponds to a fitness function that quantifies the survival success of type *x*. Types with higher-than-average fitness will proliferate; types with lower-than-average fitness will decline [53]. In contrast to Eq. (3), the general form of the replicator equation has a frequency-dependent fitness function that is the fitness ${\partial W(x,p(x,t),t)}/{\partial t}$ is a function of *p*(*x*, *t*). However, in the following we will only consider the restricted case given by Eq. (3).

In both theoretical and experimental neuroscience, there is an ongoing debate as to whether the brain directly represents uncertainty as probabilities or as log-probabilities [39, 45, 48, 82]. We are therefore also interested in the logarithmic version of Eq. (3). Introducing the new variable

4 $$\begin{aligned} U(x,t)=\log p(x,t) \end{aligned}$$

Equation (3) can be written as

5 $$\begin{aligned} \frac{\partial U(x,t)}{\partial t} = \alpha \!\left( \frac{\partial W(x,t)}{\partial t} - \sum _{x^{\prime }} \exp \left( U(x^{\prime },t) \right) \frac{\partial W(x^{\prime },t)}{\partial t} \right) .\nonumber \\ \end{aligned}$$

Here we have a simple accumulation process *U*(*x*, *t*) with inputs $\partial W(x,t)/\partial t$. The advantage of Eq. (5) is that it does not require the explicit computation of the product between *p*(*x*, *t*) and ${\partial W(x,t)}/{\partial t}$. However, it still requires computing the expectation value $\langle \partial W/\partial t\rangle _p$ that corresponds to the sum over all the products. The probability *p*(*x*, *t*) can be obtained from *U*(*x*, *t*) through the exponential transform at any point in time.

**Fig. 2:**
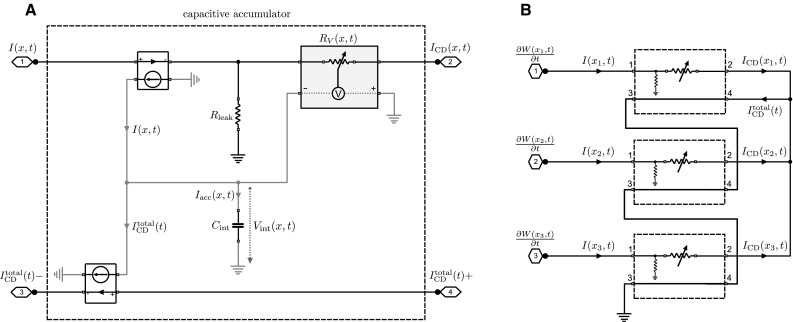
Schematic diagram of the log-space circuit with current inputs. **a** Capacitive accumulator subcircuit consisting of a primary circuit (*black wiring*) with a variable resistor that regulates the output current $I_{\mathrm {CD}}(x,t)$ and a secondary circuit (*gray wiring*) that accumulates the current difference $I(x,t)-I_\mathrm {CD}^\mathrm {total}(t)$ through a capacitor $C_\mathrm {int}$ whose voltage adjusts the variable resistor $R_{\mathrm {V}}(x,t)$. The input currents of the primary circuit are copied via current-controlled current sources to the secondary circuit. **b** Complete example circuit for three different accumulators. The individual output currents $I_\mathrm {CD}(x,t)$ are combined into $I_\mathrm {CD}^\mathrm {total}(t)$. Schematic element representations are shown in Fig. 1

### Equivalent analog circuits

In the following, we investigate two classes of bio-inspired analog circuits that implement Eqs. (5) and (3), respectively. In particular, we study differences in circuit design and the robustness properties of the circuits depending on whether uncertainty is represented in probability space or log-space. For each of the two implementations, we consider two input signal scenarios. The input signals can either be represented as currents or voltages to model different kinds of sensory encoding and to study in how far this difference in representation might lead to different circuit designs. Therefore, we consider four different circuits in the following: log-space current input, log-space voltage input, *p*-space current input and *p*-space voltage input.

A diagram of the first circuit with log-space representation and current input is shown in Fig. 2. The critical elements in the circuit are the different capacitive accumulators that integrate Eq. (5). As can be seen in Fig. 2a, each accumulator receives two input currents. The first input current *I*(*x*, *t*)—corresponding to ${\partial W}/{\partial t}$ in Eq. (5)—is specific for each accumulator. The second input current is the same for all accumulators and is given by $I_\mathrm {CD}^{\mathrm {total}}(t)$ corresponding to $\sum _{x^{\prime }} p(x^{\prime },t) {\partial W(x^{\prime },t)}/{\partial t}$ in Eq. (5). While the input current $I_\mathrm {CD}^{\mathrm {total}}(t)$ simply runs through the accumulator unaltered, the input current *I*(*x*, *t*) is fed into a *current divider* (CD—see Sect. 4 for details) with two branches, one of which connects to ground through a resistor with fixed resistance $R_{\mathrm {leak}}$, while the other branch directs current through a voltage-dependent resistor $R_{\mathrm {V}}(x,t)$ to generate the weighted output current

6 $$\begin{aligned} I_\mathrm {CD}(x,t) = \frac{R_{\mathrm {leak}}}{R_{\mathrm {leak}}+R_{\mathrm {V}}(x,t)} I(x,t). \end{aligned}$$

Figure 2b shows an exemplary full circuit with three capacitative accumulators. In the full circuit, the output currents of all accumulators as described by Eq. (6) are merged and added up to the total current

7 $$\begin{aligned} I_{\mathrm {CD}}^{\mathrm {total}} = \sum _{x^{\prime }} \underbrace{\frac{R_{\mathrm {leak}}}{R_{\mathrm {leak}}+R_{\mathrm {V}}(x^{\prime },t)}}_{ \exp (U(x^{\prime },t))} I(x^{\prime },t) . \end{aligned}$$

The total current $I_{\mathrm {CD}}^{\mathrm {total}}$ is directed as a baseline through all accumulators. Comparing Eq. (7) and the average in Eq. (5) reveals that the probability weights $p(x,t)=\exp (U(x,t))$ are given by the fraction of resistances determining the non-leaked current.

**Fig. 3:**
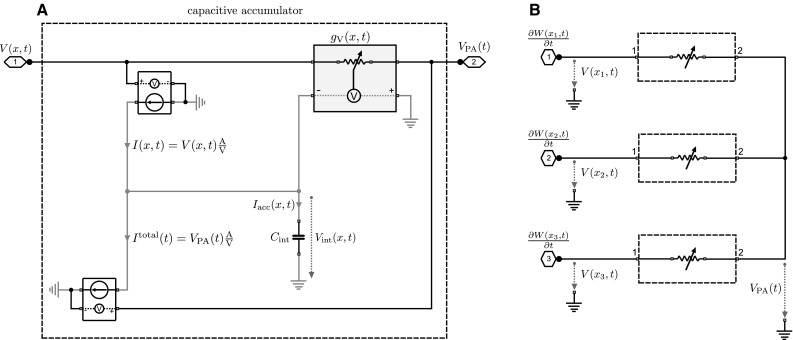
Schematic diagram of the log-space circuit with voltage inputs. **a** Capacitive accumulator subcircuit consisting of a primary circuit (*black wiring*) with a variable conductance as part of a passive averager and a secondary circuit (*gray wiring*) that accumulates the voltage difference $V(x,t)-V_\mathrm {PA}(t)$ through a capacitor $C_\mathrm {int}$ and adjusts the conductance $g_{\mathrm {V}}(x,t)$ accordingly. The voltages of the primary circuit are transformed via voltage-controlled current sources into the currents *I*(*x*, *t*) and $I^\mathrm {total}(t)$ of the secondary circuit. **b** Complete example circuit for three different accumulators. The input voltages *V*(*x*, *t*) drive the passive averager of the primary circuit to produce the weighted average voltage $V_\mathrm {PA}$. Schematic element representations are shown in Fig. 1

Inside the accumulator, the difference between the two input currents $I_\mathrm {acc}(x,t)=I(x,t)-I_{\mathrm {CD}}^{\mathrm {total}}(t)$ has to be integrated. In order to ensure that the integration process does not alter the input currents themselves by putting an extra load on the input, the integration process has to be electrically isolated, which can be achieved by generating copies of the input currents into a separate circuit. These copies can be generated by two current-controlled current sources that generate copies of the input currents $I_\mathrm {CD}^{\mathrm {total}}(t)$ and *I*(*x*, *t*), respectively. The difference between the two currents is then integrated by a capacitor with capacitance $C_\mathrm {int}$, such that

8 $$\begin{aligned} V_{\mathrm {int}}(x,t) = \frac{1}{C_\mathrm {int}} \int \left\{ I(x,t)-I_{\mathrm {CD}}^{\mathrm {total}}(t) \right\} {\hbox {d}}t . \end{aligned}$$

The voltage $V_\mathrm {int}(x,t)$ corresponds to *U*(*x*, *t*) in Eqs. (5) and (4) and the capacitance corresponds to $1/\alpha$. In line with Eq. (4), the voltage-dependent resistors $R_{\mathrm {V}}(x,t)$ depend on this voltage through an exponential characteristic line

9 $$\begin{aligned} R_{\mathrm {V}}(x,t) = R_{\mathrm {leak}} \left( \exp \left( -V_\mathrm {int}(x,t) \right) - 1 \right) . \end{aligned}$$

As long as the voltage $V_\mathrm {int}(x,t)$ represents log-probabilities and therefore assumes values between zero and negative infinity, the resistance $R_{\mathrm {V}}(x,t)$ is non-negative and well defined. Such a voltage-dependent resistor could be realized by a potentiometer with an exponential characteristic or by using the exponential relationship between current and voltage of a varistor or a transistor.

A diagram of the second circuit with log-space representation and voltage input is shown in Fig. 3. As shown in Fig. 3a, each accumulator is operated between two voltages given by the voltage *V*(*x*, *t*)—corresponding to ${\partial W}/{\partial t}$ in Eq. (5)—that is specific for the accumulator *x* and the voltage $V_{\mathrm {PA}}(t)$ that is the same for all accumulators and corresponds to the weighted average $\sum _{x^{\prime }} p(x^{\prime },t) {\partial W(x^{\prime },t)}/{\partial t}$ in Eq. (5). As the integration has to be performed by a capacitor due to the bio-inspired constraints, the voltages have to be translated into currents, which can be achieved by voltage-controlled current sources. This will also ensure that the integrator circuit is isolated as in the previous circuit and follows the same dynamics as in Eq. (8). For illustrative purposes, the diagram in Fig. 3b shows a complete circuit for three different accumulators. Essentially, the circuit corresponds to a *passive averager* (PA—see Sect. 4 for details) that combines multiple voltages each in series with a voltage-dependent conductance into a common voltage given by

10 $$\begin{aligned} V_{\mathrm {PA}}(t) = \sum _{x} \underbrace{\frac{g_{\mathrm {V}}(x,t)}{\sum _{x^{\prime }} {g_{\mathrm {V}}(x^{\prime },t)}}}_{=: p(x,t)} V(x,t). \end{aligned}$$

A comparison between Eq. (10) and the average in Eq. (5) implies that the probability weights *p*(*x*, *t*) are given by the relative conductance. To fit with Eq. (4), the voltage-dependent conductances $g_{\mathrm {V}}(x,t)$ must therefore have an exponential characteristic line

11 $$\begin{aligned} g_{\mathrm {V}}(x,t) \propto \exp \left( V_\mathrm {int}(x,t) \right) . \end{aligned}$$

In contrast to the previous circuit, the conductance $g_{\mathrm {V}}(x,t)$ is well defined for any value of the integrated voltage. In fact, changing all conductances by the same multiplicative factor does not affect the operation of the circuit.

**Fig. 4:**
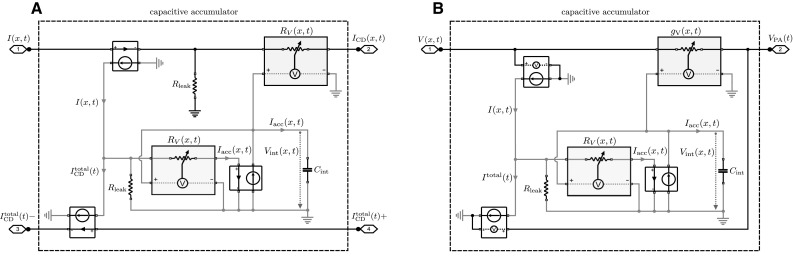
Schematic diagram of the probability space circuits. Only the accumulators are shown; example circuits are identical to Figs. 2b and 3b, respectively. **a** Capacitive accumulator subcircuit for current inputs. The accumulator consists of a primary circuit (*black wiring*) with a variable resistor and a secondary circuit that accumulates the weighted current difference $p(x,t)(I(x,t)-I_\mathrm {CD}^\mathrm {total}(t))$. The additional weighting (compared to the log-space circuit) is accomplished by an inner current divider that operates identical to the outer current divider of the primary circuit. The capacitor $C_\mathrm {int}$ integrates the weighted current difference and adjusts the resistors $R_{\mathrm {V}}(x,t)$ of the outer and inner current dividers accordingly. Another current-controlled current source is required to copy the output of the inner current divider in order to isolate the accumulation process from the rest of the circuit. **b** Capacitive accumulator subcircuit for voltage inputs. The accumulator consists of a primary circuit (*black wiring*) with a variable conductance that is critical for the passive averager and a secondary circuit (*gray wiring*) that accumulates the weighted current difference $p(x,t)(I(x,t)-I^\mathrm {total}(t))$. The additional weighting (compared to the log-space circuit) is accomplished by an inner current divider that operates identical to the current input circuit in panel A. The capacitor $C_\mathrm {int}$ integrates the weighted current difference and adjusts the conductance $g_{\mathrm {V}}(x,t)$ of the primary circuit and the resistance $R_{\mathrm {V}}(x,t)$ of the inner current divider accordingly. Schematic element representations are shown in Fig. 1

The third and the fourth circuits represent uncertainty directly in the probability space. Accordingly, they only differ from the previous circuits in terms of the inner workings of the accumulators, as the inputs $\partial W(x,t)/\partial t$ and the weighted sum $\sum _{x^{\prime }} p(x^{\prime },t) \partial W(x^{\prime },t)/\partial t$ are the same in *p*-space and log-space. Figure 4a shows an accumulator in *p*-space with external current inputs *I*(*x*, *t*). As in the first circuit, each accumulator has two inputs given by *I*(*x*, *t*) and $I_{\mathrm {CD}}^\mathrm {total}(x,t)$ and one output given by $I_\mathrm {CD}(x,t)$. As in the log-space accumulator, the output $I_\mathrm {CD}(x,t)$ corresponds to a weighted input current *p*(*x*, *t*) *I*(*x*, *t*) and this weighting is implemented by a current divider. Identical to the circuit diagram in Fig. 2b, the output currents of all accumulators are merged into the total current $I_\mathrm {CD}^\mathrm {total}(x,t)$ that is fed back as an input into the accumulators. In contrast to the log-space accumulator of the first circuit, inside the *p*-space accumulator the integral has to be taken over the *weighted* difference between the two input currents $I_\mathrm {acc}(x,t)=p(x,t) \left( I(x,t)-I_{\mathrm {CD}}^{\mathrm {total}}(t) \right)$, where the weighting with *p*(*x*, *t*) is implemented by another current divider. The voltage-dependent conductances in both current dividers have to be adjusted according to

12 $$\begin{aligned} R_{\mathrm {V}}(x,t) = R_{\mathrm {leak}} \left( \frac{1}{V_\mathrm {int}(x,t)} -1 \right) , \end{aligned}$$

as the voltage $V_\mathrm {int}$ now directly represents *p*(*x*, *t*) and therefore only assumes values in the unit interval. Note that the leak resistances $R_{\mathrm {leak}}$ in the two current dividers of each accumulator do not have to be identical, but the variable conductances always have to be adjusted such that the equality

13 $$\begin{aligned} \frac{R_{\mathrm {leak}}}{R_{\mathrm {leak}}+R_{\mathrm {V}}(x,t)} = V_\mathrm {int}(x,t) = p(x,t) \end{aligned}$$

holds for each current divider. The weighted current of the inner current divider $I_\mathrm {acc}(x,t)$ is copied by a current-controlled current source and then integrated as a voltage over $C_\mathrm {int}$. The same voltage $V_\mathrm {int}(x,t)$ over the capacitance has to drive both voltage-dependent conductances in both current dividers in order to implement Eq. (3).

Figure 4b shows an accumulator in *p*-space where the external inputs are given as voltages *V*(*x*, *t*). As in the second circuit, each accumulator is operated between the two voltages *V*(*x*, *t*) and $V_\mathrm {PA}(x,t)$. Identical to the circuit diagram in Fig. 3b, voltage $V_\mathrm {PA}$ is determined by a passive averager across the different accumulators. As in the second circuit, the voltages are transformed into currents when they enter the accumulators by voltage-controlled current sources. The important difference to the log-space accumulator of the second circuit is again that in *p*-space the integral has to be taken over the *weighted* difference between the two currents $I_\mathrm {acc}(x,t)=p(x,t) \left( I(x,t)-I^{\mathrm {total}}(t) \right)$. This weighting is implemented by a current divider in an identical fashion as in the previous circuit shown in Fig. 4a. In this case, the voltage-dependent conductance of the current divider follows Eq. (12) and the voltage-dependent conductance of the passive averager follows a simple proportionality characteristic given by $g_{\mathrm {V}}(x,t) \propto V_\mathrm {int}(x,t)$.

### Simulations

**Fig. 5:**
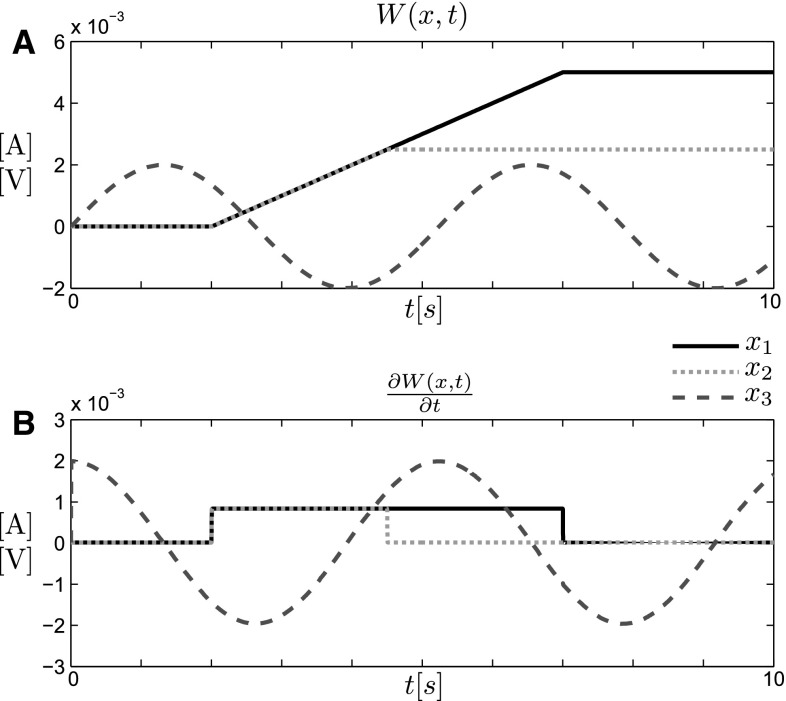
Input signals for simulation. **a** Time course of the three integrated signals *W*(*x*, *t*) indexed by $x \in \{ x_1,x_2,x_3\}$. The signals represent the evidence of a particular hypothesis or the utility of a particular action. For $x_1$ and $x_2$, the evidence or utility grows with a constant rate until saturation is reached. For $x_3$, the evidence or utility is waxing and waning, following a sine function. *Note* that higher values correspond to more probable hypotheses or more desirable actions. A rational decision-maker following Eq. (19) should thus initially favor $x_3$. After the onset of $x_1$ and $x_2$, the decision-maker should be indifferent between these two options but should disfavor $x_3$. After $x_2$ reaches saturation, $x_1$ should be favored. **b** Inputs $\partial W(x,t)/\partial t$ which are fed into the circuits as either currents or voltages and drive the competitive integration process

To test the noise robustness of the circuits shown in Figs. 2, 3, 4, we simulated their dynamics in a Simulink$^{\circledR }$ environment with idealized components, which we could selectively perturb by band-limited white noise. Put simply, these simulations are trying to reproduce competition between different streams encoding the evidence for alternative hypotheses without violating the obvious requirement that the resulting probabilities should sum to one. In our examples, we simulated three different time-varying inputs $\partial W(x_i,t)/\partial t$ indexed by $x \in \{x_1,x_2,x_3\}$. The first input was a rectangular pulse of 5 s with an amplitude of $10^{-3}$ A in the circuits with current-based inputs and $10^{-3}$ V in the circuits with voltage-based inputs. The second input was a rectangular pulse of 2.5 s with the same amplitude. The third input was a cosine with amplitude $2 \times 10^{-3}$ A (or V) and a frequency of 0.19 Hz. The first two inputs mimic the more usual scenario where evidence is increased over a particular time window at a constant rate, whereas the third input is the more unusual scenario with waxing and waning evidence. The first two inputs are integrated into a ramp with different plateaus, and the third input integrates into a sine wave. The input signals and their integrals are shown in Fig. 5.

**Fig. 6:**
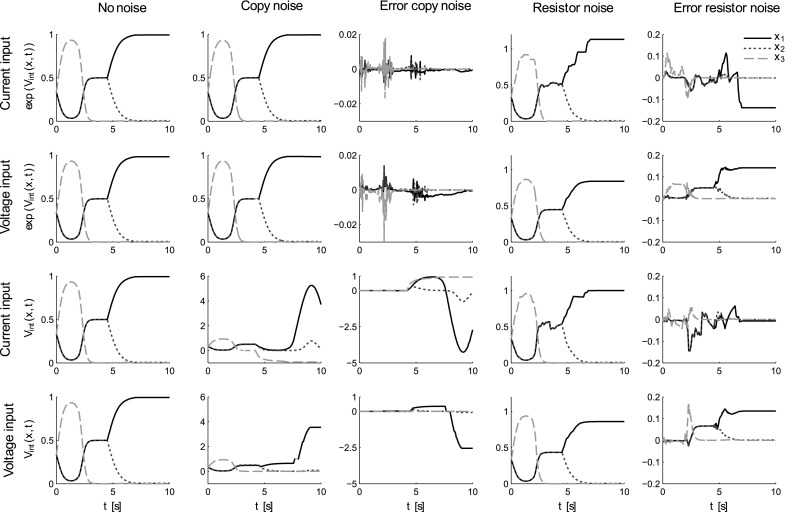
Robustness simulation for all circuits. The *top two rows* show the simulated probabilities $p(x,t)=\exp (V_\mathrm {int}(x,t))$ for the log-space circuits—*first row* current input, *second row* voltage input. The *bottom two rows* show the simulated probabilities $p(x,t)=V_\mathrm {int}(x,t)$ for the probability space circuits—*first row* current input, *second row* voltage input. The *first column* shows the results for a noise-free simulation, where all circuits perform identically and consistent with the replicator equation. The *second column* shows the results where band-limited white noise was injected into the copy elements, that is, the current- or voltage-controlled current sources. The magnitude of the noise was identical for all circuits. The *errors* with respect to the corresponding noise-free simulation are shown in the third column. The *fourth column* shows the results where band-limited white noise was injected into the voltage-dependent resistors. Again the magnitude of the noise was identical for all circuits. The *errors* with respect to the corresponding noise-free simulation are shown in the last column

We simulated all four circuits shown in Figs. 2, 3, 4 under three noise conditions. As a baseline, we first simulated all circuits without noise and plotted the probability encoded by the voltage of the integrating capacitors. In case of the log-space circuits, this corresponds to the exponential of the voltage. In the p-space circuits, the voltage directly encodes probability. This can be seen in the first column of Fig. 6. In the first 2 s, the cosine signal has the highest amplitude and therefore the highest probability weight, before it is overtaken by the onset of the two pulses. Eventually, the longer lasting pulse dominates the probability weighting.

In the second noise condition, we added band-limited white noise on the output currents of all copying elements, that is, all voltage- or current-controlled current sources. The standard deviation of the noise was $1\,\mathrm {\upmu A}$ and roughly corresponded to three orders of magnitude below the maximum input signal. As can be seen from the simulation in the second and third column of Fig. 6, the noise has very different effects in the *p*-space and log-space circuits. While the log-space circuits show errors on the order of percentages in probability space, the p-space circuits fail and completely leave the range of permissible probability values. The circuit element in the *p*-space circuit that is responsible for this failure is the current-controlled current source that directly feeds into the integrator. The ultimate reason for this difference is of course that the integrator voltage in the *p*-space circuit is confined between zero and one, whereas the integrator voltage in the log-space circuit can take any negative value.

In the third noise condition, we added band-limited white noise on the resistance of the voltage-dependent conductances. The standard deviation of the noise was $50\,\varOmega$. According to Eqs. (9) and (12), the voltage-dependent resistance in the circuits with current inputs can decrease to almost zero for dominant inputs, but can take on values up to $10^8\,\varOmega$ in our simulations. In contrast, the voltage-dependent resistance in the circuits with voltage inputs do not have to regulate their resistance down to zero, but to an arbitrary baseline resistance—because this baseline resistance cancels out in the passive averager. Accordingly, one would expect the most disruptive effects of noise for dominant inputs with high probability weighting, but less so in the case of passive averager circuits that operate on voltage inputs. In Fig. 6, it can be seen that the noise-corrupted probabilities in the passive averager circuits are much smoother for high probability weightings than in the current divider circuits. However, there seems to be no difference in the magnitude of the errors.

**Fig. 7:**
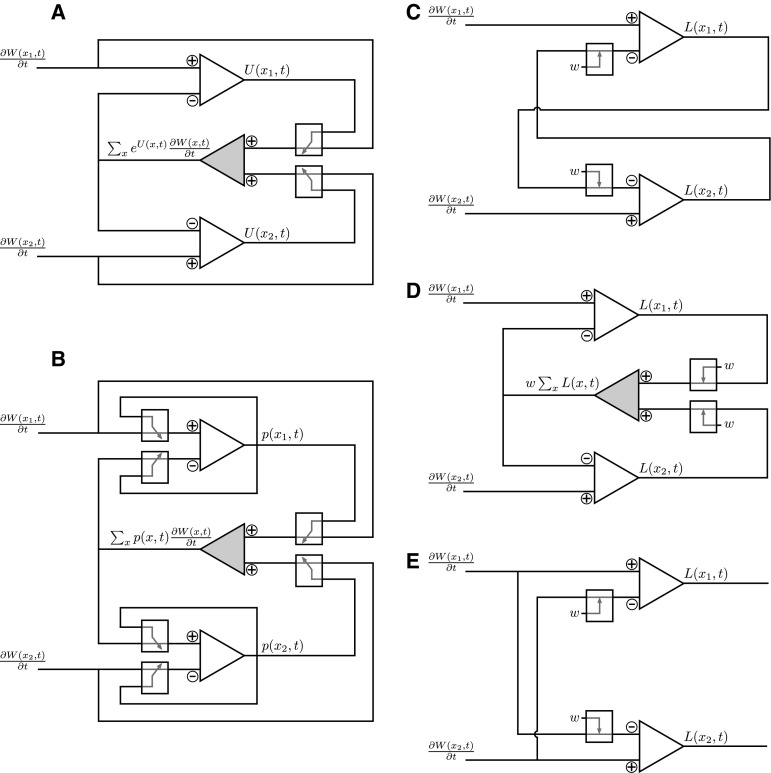
Neural circuit diagrams for competitive signal integration. *White triangles* represent excitatory units corresponding to different accumulators *x*. *Gray triangles* correspond to inhibitory units. **a** Replicator dynamics in log-space according to Eq. (5). The *little boxes* with *arrows* denote a multiplicative modulation with an exponential characteristic. **b** Replicator dynamics in probability space according to Eq. (3). The *little boxes* with *arrows* denote a multiplicative modulation. **c** Mutual inhibition circuit. The *little boxes* with *arrows* denote a fixed weighting. **d** Pooled inhibition circuit. The *little boxes* with *arrows* denote a fixed weighting. **e** Feed-forward inhibition. The *little boxes* with *arrows* denote a fixed weighting

### Implications for possible neural circuits

As already mentioned, there is an ongoing debate about whether the brain directly represents uncertainty as probabilities or as surprise, that is, log-probabilities. In the previous section, we have considered both possibilities in different circuit designs. As illustrated in Fig. 7, these bio-inspired analog circuit designs can serve as abstract templates for schematic neural circuits. Figure 7a shows a free energy optimizing neural circuit operating in log-space—compare Eq. (5). Input signals are excitatory and integrated by accumulator neurons that are inhibited at the same time by a pooled inhibition signal. To establish this inhibition signal, copies of all inputs are summed up by an inhibitory neuron that sends its signal to all accumulator neurons. The most critical operation in this circuit would require that the output signal *U* of the accumulator neuron modulates the weighting of the input signal $\partial W/\partial t$ before it enters the inhibitory unit. Moreover, this modulation of the input signal would have to correspond to a multiplicative weighting where the weighting factor is characterized by an exponential dependency on the excitatory output signal, such that the modulated input to the inhibitory neuron is given by $e^{U} \partial W/\partial t$. As *U* is the log-probability and therefore always negative, the weighting factor $e^U$ could also be interpreted in terms of a synaptic transmission probability that is modulated by the signal *U*.

Figure 7b shows a schematic of a free energy optimizing neural circuit in probability space—compare Eq. (3). The basic principle of the circuit is the same as in Fig. 7a. The important differences between the *p*-space and log-space neural circuits are the following. First, the output of the accumulator neurons represents a probability *p* instead of the log-probability *U*. Second, each accumulator modulates its own inputs by a multiplicative factor given by the output activity *p*—this concerns both the excitatory input $\partial W/\partial t$ and the inhibitory input $<\partial W/\partial t>_p$. Third, all multiplicative modulations are characterized by weighting factors that are proportional in *p* and not exponential as in the log-space case. Overall, the *p*-space circuit is more complex with nested recurrencies that require the simultaneous modulation of multiple sites in dependence of the same signal *p*.

In the literature, the dynamics of neural circuits for competitive signal integration are often modeled by drift diffusion processes [8, 9, 12, 35]. In these models, momentary evidence modulates the drift in a Brownian motion process. Mainly, four different kinds of drift diffusion models are distinguished: race models [75], mutual inhibition models [74, 79], feed-forward inhibition models [47, 65] and pooled inhibition models [77, 80]. Race models consist of independent accumulators without any inhibitory interactions and can therefore be disregarded in this context. We consider the other three inhibition models in the following. Linearized mutual inhibition models may be described by the dynamics

14 $$\begin{aligned} \dot{L}_i = - k L_i + I_i - w \sum _{j \ne i} L_j , \end{aligned}$$

where $x_i$ denotes activity of accumulator *i*, *k* is a self-inhibition factor, *w* is the inhibitory weighting factor between the different neurons and $I_i$ is the input signal. The corresponding circuit is shown in Fig. 7c. Similarly, one can express the simplified dynamics of a pooled inhibition model as

15 $$\begin{aligned} \dot{L}_i = - k L_i + I_i - w \sum _{j} L_j , \end{aligned}$$

where all neurons contribute equally to the global inhibitory signal. The corresponding circuit is shown in Fig. 7d. In contrast, feed-forward inhibition models only modulate their activity depending on the inputs *I*, such that

16 $$\begin{aligned} \dot{L}_i = - k L_i + I_i - w \sum _{j \ne i} I_j , \end{aligned}$$

where *w* indicates the inhibitory effect of input $I_j$ on accumulator *i*. In this case, each input has connections with all accumulators, of which all but one are inhibitory. The corresponding circuit is shown in Fig. 7e.

As the input only enters additively in Eqs. (14)–(16), it makes sense to compare these models to the log-space circuit in Fig. 7a. The most obvious difference of the log-space circuit in Fig. 7a from all the other circuits listed above is that inhibition depends on both the inputs *I* and the neural activity *U* such that

17 $$\begin{aligned} \dot{U}_i = I_i - \sum _{j} e^{U_j} I_j . \end{aligned}$$

There is no self-inhibition in these dynamics, as the introduction of a decay term $-k L_i$ would compromise the normalization $\sum _i p_i = 1$ of $p_i = \exp (U_i)$. Note that none of the other accumulators is normalized and therefore a separate normalization step is required. Comparing Eqs. (17)–(14), (15) and (16) raises the question of how accumulator dynamics of Eqs. (14)–(16) could approximate dynamics of the form of Eq. (17) that are required for Bayesian inference and bounded rational decision-making.

Important differences between the dynamics of Eqs. (14)–(17) can be illustrated by considering constant inputs *I*. For constant inputs, Eqs. (14)–(16) reach steady-state attractors where $\dot{L_i} = 0$ for all *i*. In contrast to these three inhibition models, update Eq. (17) does not reach a steady state unless all inputs are the same, that is, $I_i = I_j \,\,\forall i,j$. This is the case, for example, when all hypotheses have the same likelihood or when all actions lead to the same increase in utility and therefore the posterior probabilities simply equal the prior probabilities. If the inputs are not the same in Eq. (17), we have the limit behavior $U_i \rightarrow 0$ if $i=\arg \max _i I_i$ otherwise $U_i \rightarrow -\infty$. The exponential $\exp (U_i)$ is always bounded by one of the asymptotes 0 or 1. This difference between the models with respect to their limit behavior originates from the presence or absence of the decay term $-k L_i$. If this term is omitted in the other models, the $L_i$ can also grow without bound both in the positive and negative direction. However, there are also important differences between the models even if we disregard the decay term $-k L_i$. For constant inputs, the mutual inhibition model exhibits exponential growth. In contrast, the feed-forward inhibition model always has a constant growth rate and the pooled inhibition model converges to constant growth rates. The free energy update Eq. (17) also converges to constant growth rates and is therefore qualitatively most similar to the pooled inhibition model. However, both their modulation of the growth rates through the dynamics of $L_i$ and $U_i$ before convergence and the limit values of $L_i$ and $U_i$ differ.

Here we have focused on evidence accumulation with a finite number of accumulators where each accumulator corresponds to a different hypothesis. This corresponds to the scenario that is usually considered by evidence accumulation models based on drift diffusion processes [9, 49]. An obvious question is of course how to generalize this kind of setup to continuous hypothesis spaces. For particular families of distributions, like for example Gaussian distributions, one can replace an infinite number of accumulators by a finite number of sufficient statistics, for example mean and variance in the case of the Gaussian. This is exploited for example in Kalman filters and some predictive coding models [3, 26, 62]. Other possibilities include representing uncertainty through gain encoding or through convolutional codes with a finite number of basis functions [39]. Due to the many possibilities how one could think about a continuous generalization, we restrict ourselves to discrete states in the current study.

## Discussion

In this study, we have described four bio-inspired analog circuit designs implementing the frequency-independent replicator equation. The frequency-independent replicator equation optimizes a free energy functional and can be used to describe both competitive evidence accumulation for perception and utility accumulation for action. The bio-inspired circuits differed in whether they implemented the frequency-independent replicator equation directly in probability space or in log-probability space and in whether the input signal was given as a voltage or as a current. The circuits were designed under the constraint that they should only consist of a restricted set of electrical components that are biologically interpretable in the sense that such components are commonly used when neural circuitry is schematized by equivalent electrical circuits. Accordingly, we sketch how the two basic circuit designs for free energy optimization in probability and log-probability space might translate into neural wiring in Fig. 7. Here we discuss the biological plausibility of these circuits.

### Biological plausibility

In standard textbooks [51], neurons are usually modeled as capacitors that integrate currents over time and that have synapses and ion channels that can change their conductance depending on voltage. Also the neural integrators in our circuits are modeled as capacitors. The basic design of the circuits in Fig. 7 implies that each neural integrator receives both excitatory and inhibitory inputs. As all neural integrators receive the same inhibitory input, it is natural to assume that the inhibitory signals stem from a single inhibitory unit that pools copies of all the excitatory inputs and feeds the resulting inhibitory signal back to the neural integrators. This would imply, however, that inhibitory neurons mainly perform a spatial integration, whereas excitatory neurons would mainly perform a temporal integration. Accordingly, the inhibitory neurons would have to compute their output quasi-instantaneously compared to the time scales of the input. This very same problem is faced by all pooled inhibition models. Here the particular challenge of the circuit diagrams shown in Fig. 7 is the temporal dependence of the weights for averaging, as the probability weights would have to change on the same time scale as the inhibitory output activity that is quasi-instantaneously with respect to the time scales of the input signal.

As already described in the previous section, the critical operation in the free energy circuits is performed by the voltage-dependent conductances that regulate how much of any particular input signal reaches the inhibitory unit. In particular, in the log-space circuits it would be required that there is an exponential relation between the voltage signal and the resulting conductance or transmission probability. This would be a very particular property to look for in possible neural substrates. In contrast, this exponential relationship is not required in the p-space circuits. However, their biological plausibility suffers from two other deficiencies. First, the circuit design is considerably more complex than the log-space circuit design in that it requires multiple replications of the same voltage-dependent conductances that not only modulate the inputs to the inhibitory units, but also the inputs to the excitatory neural integrators. Second, as is evident from the simulations, the p-space circuits are extremely susceptible to noise.

Another implementation challenge of Eq. (5) is that the most unlikely hypotheses or actions require the accumulation signal *U* with the strongest magnitude that is the highest currents and the highest voltages. This is a natural consequence of operating in the log-domain, where unexpected events are assigned the most resource-intense encoding, such that expected events can be encoded more efficiently [46]. However, when implementing Eq. (5) in a real physical system this problem could be solved naturally, as any physical signal will have a natural minimum and maximum that is technically feasible. For example, if the physical signal that is used for representation of $U_i$ has a natural limit between zero and a minimum $-M=-\log (L)$, that is $U_i \in [-M; 0]$, then the probability $p_i = \exp (U_i)$ is confined to the interval $\frac{1}{L} \le p_i \le 1$ with a minimum nonzero probability 1 / *L* assigned to any hypothesis or action. In order to deal with exclusively positive signal ranges, one can also redefine the representation as $x_i = \log p_i + \log L$ which implies $0 \le x_i \le \log (L)$. This redefined representation has the convenient effect that improbable hypotheses or actions are no more associated with the highest signal magnitude, but with the lowest. Similar cutoffs in the precision of probability representations are ubiquitous in Bayesian statistics, for example in the context of Cromwell’s rule or Occam’s Window.

### Divisive normalization

As an alternative to modeling a single process that accomplishes signal integration and competition simultaneously, one could imagine a model where signal integration and competition are dealt with in separate stages of the process or even as two separate processes or mechanisms. The integration process does not pose any particular problem, but simply corresponds to independent integration processes of individual excitatory signals. In an analog circuit, this would correspond as usual to a capacitor that integrates currents into a voltage. The competition between the different integrated signals can then be introduced after integration by the application of a softmax function

18 $$\begin{aligned} p(x,t) = \frac{\exp \left( \alpha W(x,t)\right) }{\sum _{x^{\prime }}\exp \left( \alpha W(x^{\prime },t)\right) } , \end{aligned}$$

where $\alpha$ is the same temperature parameter as in Eq. (1) and *W*(*x*, *t*) is the integrated signal $\int \partial W$. This is the mathematical operation of divisive normalization. For example, Bayesian inference could be achieved in log-space by such a two-step process, where first log-likelihoods are integrated or added up over time and in a second step the summed or integrated signals are squashed through a softmax function [48]. Importantly, Eq. (18) optimizes the free energy functional of Eq. (2) under uniform priors. Nonuniform priors can be included to yield

19 $$\begin{aligned} p(x,t) = \frac{p(x,t=0) \exp \left( \alpha W(x,t)\right) }{\sum _{x^{\prime }} p(x^{\prime },t=0) \exp \left( \alpha W(x^{\prime },t)\right) } , \end{aligned}$$

where $\log p(x,t=0)$ can be interpreted as the initial state of the accumulator *x*. While there exist analog implementations of the softmax function [24, 40, 83], these implementations have a circuit design that is not easily interpretable in terms of equivalent neural circuits. For example, the circuits in [24] enforce a constant output current that is additively composed of drain currents from multiple transistors that are controlled by exponentially weighted gate voltages. The softmax function is computed by the individual drain currents. However, in a biological setting a constant output current that drives the integration process is not plausible. Nevertheless, other implementations might be possible.

In neuroscience, divisive normalization has been advanced as a fundamental neural computation over the last two decades [14]. It has been suggested as a normalization mechanism to regulate stimulus sensitivity in the invertebrate olfactory system [54], the mammalian retina [52], primary visual cortex [13, 15] and other cortical areas [32]. However, the biophysical mechanisms and possible circuit designs that would support divisive normalization are still under debate [14]. One of the earliest proposed mechanisms for divisive normalization is shunting inhibition mediated by synapses that cause a change in membrane conductance without a major change in current flow [63]. For constant input, however, shunting only has a divisive effect on the membrane potential in integrate-and-fire models of neural activity, but not on the firing rate of these neurons [33]. This has led to the more recent proposal that shunting might be achieved by temporally varying changes in conductance [16, 69]. However, physiological evidence for this mechanism remains mixed [14]. Other proposed physiological mechanisms that could mimic divisive normalization at least for some experimental data are synaptic depression and modulation of ongoing activity to keep membrane potentials closer or further from the spiking threshold [1, 64]. As divisive normalization seems to play such a prominent role in biological information processing, our circuits might inspire an interesting alternative that does not require a separate mechanism for normalization, but a single process that automatically generates normalized signals. However, as discussed in the previous section the biological plausibility of these circuits is certainly also open for debate.

### Circuits for Bayesian integration

Several hardware implementations of inference processes have been proposed in the recent past [41, 50, 81]. The implementation of continuous-time Bayesian inference in analog CMOS circuits, for example, has been recently discussed by Mroszczyk and Dudek [50]. The authors investigate message passing inference schemes in Bayesian networks that consist of multiple variables that factorize. The analog implementation they propose is based on the Gilbert multiplier that is seconded by transistor circuits such that the overall multiplier circuit can normalize incoming current signals. While these circuits are technologically optimized for accuracy and scalability, the building blocks of these circuits make a biological interpretation difficult. At the other end of the spectrum of biological realism, VLSI implementations of spiking neural networks for real-time inference have been recently proposed [18]. In contrast, the current study does not reach the neuromorphic realism of spiking networks, but starts out by addressing the question of how free energy optimizing dynamics could be implemented in circuits that allow for some degree of biological interpretation. While the direct implementation of such circuits seems to have received little attention so far, some special cases of the general replicator equation that correspond to the Lottka–Volterra equation have been implemented in VLSI to better understand competitive neural networks [2]. However, the equivalence between the replicator equation and the Lottka–Volterra equations does not hold for the frequency-independent replicator equation and therefore does not concern our results.

Bayesian inference has been proposed as a fundamental theory of perception and a considerable number of different neural implementations of inference processes have been proposed in the recent past [4, 21, 39, 43, 44, 61]. However, one might regard Bayesian inference as a particular instantiation of a more abstract optimization principle given by the free energy difference in Eq. (2), when the utility is given by a log-likelihood [25, 58]. Intriguingly, the same principle can be generalized to the problem of acting. A decision-maker starts out with a prior strategy and considers different options with different utilities. When the set of options is large, it might be impossible to consider all of them, such that the decision-maker has to make a decision after sampling a few possibilities [56, 59]. Making a decision based on these samples, the decision-maker effectively follows a probabilistic strategy that can be described by the posterior distribution in Eq. (1) optimizing a trade-off between utility gain and computational cost. The computational cost is measured by the relative entropy between prior and posterior strategy. Compared to a perfect decision-maker, such a decision-maker is bounded rational since he can only afford a limited amount of information processing. The principle issues of the proposed circuitries might therefore be applicable both to perception and action.

One of the main problems of implementing Bayesian integration is the issue of tractability, which often arises due to the computation of the normalization constant, especially when integrating over high-dimensional parameter spaces, but also, for example when summing over discrete states in larger size undirected graphical models. One way to deal with this kind of problem is to investigate stochastic and sampling-based approximations of probabilistic update schemes [21, 28, 34, 60, 67, 68]. Here we were not primarily interested in such stochastic implementations, because like many previous studies we were interested in circuits that integrate a finite number of given inputs and do not probabilistically ignore some inputs. Naturally, our circuits then do not provide a generic solution to Bayesian inference in arbitrary networks, but rather we have restricted ourselves to the special case of competitive evidence accumulation with a finite number of given inputs. If such input streams are given in terms of physical signals, then computing a weighted average by summing over these signals is certainly a tractable operation. Even though such competitive signal integration is equivalent to a Bayesian inference process [8], if one were interested in generic Bayesian inference in possibly continuous and high-dimensional parameter spaces, one would certainly need to consider some kind of approximation to Eqs. (1) and (3)—see for example [56] for a sampling-based implementation.

### Circuits for free energy optimization

Free energy optimization has been studied previously in Hopfield networks in the context of memory retrieval and in Boltzmann machines in the context of learning generative probabilistic models. Both Hopfield networks and Boltzmann machines can be described by the same kind of energy function; only the dynamics of the latter are stochastic. The energy function

$$\begin{aligned} E[s] = -\frac{1}{2} \sum _{i,j} w_{ij} s_i s_j - \sum _i b_i s_i \end{aligned}$$

specifies the desirability of the binary state $s=\{s_1, \ldots , s_n\}$ of all neurons *i* in the network with $s_i \in \{-1,+1\}$ under given parameters $w_{ij}$ and $b_i$. In both networks, the dynamics $s_t \rightarrow s_{t+1}$ minimize this energy function, which corresponds to a relaxation process into an equilibrium distribution $p_{\mathrm {eq}}(s)$ over states *s*. Thus, the free energy does not play a direct role in the dynamics. However, if one restricts the class of equilibrium distributions to special classes of parameterized separable distributions $p_{\theta }(s)$, then one can optimize the variational free energy

$$\begin{aligned} F(\theta ) = \sum _{s} p_{\theta }(s) E[s] + \sum _{s} p_{\theta }(s) \log p_{\theta }(s) \end{aligned}$$

to find the distribution $p_{\theta }(s)$ that most closely matches the equilibrium distribution $p_{\mathrm {eq}}(s)$. In the case of Hopfield networks, this leads for example to a mean field approximation—compare Chapter 42 in [46].

Apart from the dynamics that govern the state evolution in these networks, there are also update rules that determine the parametric weights $w_{ij}$ and $b_i$ of the networks during learning [6]. In Boltzmann machines with hidden units *h*, the equilibrium distribution over observable states *x* is given by $p_{\mathrm {eq}}(x)=\sum _{h} e^{-E[(x,h)]} / Z_E$ where $s=(x,h)$ and $Z_E$ is a normalization constant. Using the free energy

$$\begin{aligned} F(x) = - \log \sum _{h} e^{-E[(x,h)]} \end{aligned}$$

the equilibrium distribution can be expressed as a Boltzmann distribution $p_{\mathrm {eq}}(x) = e^{-F(x)} / Z_F$ with normalization constant $Z_F$. Learning a generative model for *x* can then be achieved by updating the parameters $w_{ij}$ with the gradient

$$\begin{aligned} \frac{\partial \log p_{\mathrm {eq}}(x)}{\partial w_{ij}} = - \frac{\partial F(x)}{\partial w_{ij}} + \frac{\partial }{\partial w_{ij}} \log Z_F \end{aligned}$$

and similarly for the parameters $b_i$. Crucially, however, such learning updates change the energy function itself by optimizing the log-likelihood of the data. Challenges of physical implementations of Boltzmann machines have been discussed in [23].

**Fig. 8:**
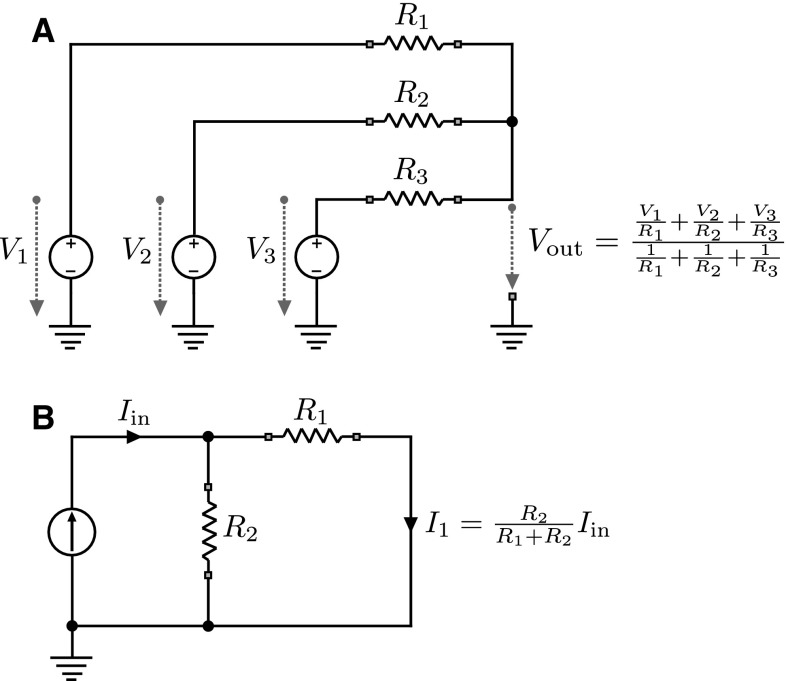
**a** Passive averager (PA) circuit. The output voltage $V_\mathrm {out}$ is the weighted average of the voltages $V_1, V_2$ and $V_3$, where the weights are given by the resistors $R_1, R_2$ and $R_3$. **b** Current divider (CD) circuit. The input current $I_\mathrm {in}$ is divided into two currents over the two resistors $R_1$ and $R_2$ where the magnitude of the current that flows through each branch is proportional to the conductance of each branch

Both kinds of free energy updates are not directly relevant to our study, as neither the Hopfield network nor the Boltzmann machine can be used to optimize arbitrary free energy functions for competitive evidence accumulation. Both network types have been designed to solve completely different problems—i.e., memory retrieval and generative model learning. The energy function in these networks describes a particular recurrent network dynamics and does not constitute an external signal that is integrated over time. In contrast, in our circuits we study possible implementations of the evolution of the posterior distribution for decision-making or inference resulting from the temporal integration of a time-dependent external input signal. Unlike the distributions in Hopfield and Boltzmann machines that relax to equilibrium, the posterior in our implementations is an equilibrium distribution at any point in time as long as it follows the dynamics of Eq. (3). In any physical implementation, this can of course only be approximately true as long as the time scale of the input signal is slow compared to physical delays, etc. In the future, it might therefore also be interesting to study non-equilibrium systems for decision-making and inference [30].

## Materials and methods

All simulations were performed using the Simscape$^{\mathrm{TM}}$ library of MATLAB$^{\circledR }$ R2012b Simulink$^{\circledR }$. We simulated all circuits using the numerical solver *ode15s*.

In the log-space circuit with current inputs shown in Fig. 2, the weighting with *p*(*x*, *t*) is performed by using current dividers (CD) with variable resistors. A schematic diagram of the basic current divider principle is shown in Fig. 8b. To compute the weighted average $I_\mathrm {CD}^\mathrm {total}(t)$ as given by Eq. (7), each input current *I*(*x*, *t*) in Fig. 2b is fed into a current divider that outputs the weighted current $I_\mathrm {CD}(x,t)=p(x,t)I(x,t)$. These currents are then summed up by connecting the current dividers into a common point which produces $I_\mathrm {CD}^\mathrm {total}(t)$. To ensure proper operation of the circuit, the voltage-dependent resistors of the current dividers have to be precisely set according to Eq. (9). To simulate the log-space circuit with current inputs shown in Fig. 2, we used the following components. We set $C_{\mathrm {int}}=500\,\upmu$F for the integrators which corresponds to $\alpha =2$ given the magnitudes of the input currents shown in Fig. 5. The integrator capacitors are initialized with $V_\mathrm {int}(x,t=0)= -\ln 3 \mathrm {V} \approx -1.0986$ V corresponding to $p(x,t=0)=1/3$. The voltage-dependent resistor is simulated as

20 $$\begin{aligned} R_{\mathrm {V}}(x,t)=R_{\mathrm {leak}}(\exp (V_\mathrm {int}(x,t)/1\mathrm {V})-1), \end{aligned}$$

with $R_{\mathrm {leak}}=100\,\varOmega$ for the fixed resistor of the current divider.

The probability space circuit with current inputs shown in Fig. 4a is very similar to the log-space implementation of Fig. 2. The major distinction is that in the probability space circuit the difference $I(x,t)-I_\mathrm {CD}^\mathrm {total}(t)$ is weighted with *p*(*x*, *t*) to form the accumulated current $I_\mathrm {acc}$, whereas in the log-space circuit the difference is directly integrated without an additional weighting. The additional weighting in the probability space circuit is accomplished with an inner current divider that operates identical to the outer current divider, both of which are adjusted according to Eq. (12):

21 $$\begin{aligned} R_{\mathrm {V}}(x,t)=R_{\mathrm {leak}}\left( \frac{1}{V_\mathrm {int}(x,t)}1\mathrm {V}-1\right) , \end{aligned}$$

with $R_{\mathrm {leak}}=100\,\varOmega$ for the fixed resistors of the current dividers. Note that in the probability space circuits $V\mathrm {int}(x,t)$ directly corresponds to *p*(*x*, *t*). Therefore, the integrator capacitors are initialized with $V_\mathrm {int}(x,t=0)= 1/3 \mathrm {V}$ corresponding to $p(x,t=0)=1/3$. The capacitance $C_{\mathrm {int}}=500\,\upmu$F is set as in the previous circuit.

In the log-space circuit with voltage inputs shown in Fig. 3, the weighting with *p*(*x*, *t*) and summation over all *x* are performed simultaneously by using a passive averager (PA) with variable conductances. A schematic diagram of the basic passive averager principle is shown in Fig. 8a. To compute the weighted average $V_\mathrm {PA}(t)$ as given by Eq. (10), the input voltages *V*(*x*, *t*) in Fig. 3b are combined through a passive averager that produces the weighted voltage $V_\mathrm {PA}(x,t)=\sum _{x^{\prime }} p(x^{\prime },t)V(x^{\prime },t)$. To ensure proper operation of the circuit, the voltage-dependent conductances of the passive averager have to be precisely set according to Eq. (11). To simulate the log-space circuit with voltage inputs shown in Fig. 3, we used the following components. We set $C_{\mathrm {int}}=500\,\upmu$F for the integrators which corresponds to $\alpha =2$ given the magnitudes of the input currents shown in Fig. 5. The integrator capacitors are initialized with $V_\mathrm {int}(x,t=0)= -\ln 3 \mathrm {V} \approx -1.0986$ V corresponding to $p(x,t=0)=1/3$. The voltage-dependent conductances are simulated as

22 $$\begin{aligned} g_{\mathrm {V}}(x,t)=100 \varOmega ~\exp (V_\mathrm {int}(x,t) / 1\mathrm {V}) . \end{aligned}$$

The probability space circuit with voltage inputs shown in Fig. 4b is very similar to the log-space implementation of Fig. 3. The major distinction is that in the probability space circuit the difference $I(x,t)-I^\mathrm {total}(t)$ is weighted with *p*(*x*, *t*) to form the accumulated current $I_\mathrm {acc}$, whereas in the log-space circuit the difference is directly integrated without an additional weighting. The additional weighting in the probability space circuit is accomplished with an inner current divider that operates identical to the inner current divider of the probability space circuit with current input and is adjusted according to Eq. (12):

23 $$\begin{aligned} R_{\mathrm {V}}(x,t)=R_{\mathrm {leak}}\left( \frac{1}{V_\mathrm {int}(x,t)}1\mathrm {V}-1\right) , \end{aligned}$$

with $R_{\mathrm {leak}}=100\,\varOmega$ for the fixed resistor of the current divider. The outer weighting operation is performed with a passive averager, and thus, the voltage-dependent conductances have to be set according to:

24 $$\begin{aligned} g_{\mathrm {V}}(x,t)=100 \varOmega ~V_\mathrm {int}(x,t) / 1\mathrm {V} . \end{aligned}$$

Note that in the probability space circuits $V\mathrm {int}(x,t)$ directly corresponds to *p*(*x*, *t*). Therefore, the integrator capacitors are initialized with $V_\mathrm {int}(x,t=0)= 1/3\, \mathrm {V}$ corresponding to $p(x,t=0)=1/3$. The capacitance $C_{\mathrm {int}}=500\,\upmu$F is set as in the previous circuit.

In order to illustrate the robustness of the circuits against perturbations, we injected noise into the copying elements—i.e., the voltage- and current-controlled current sources—and into the voltage-dependent resistors or conductances. As a noise source, we used the Simulink$^{\circledR }$ band-limited white noise block which allows to introduce band-limited white noise into a continuous system. We set the parameters of the block to the following values: Noise Power $=0.1$ and Sample Time $=0.1\,\hbox {s}$ (see Simulink$^{\circledR }$ documentation for more information). Additionally, we scaled the output of the white noise source with a constant multiplicative factor. In order to inject noise into the copy elements we controlled a current source with the white noise block and a scaling factor of $10^{-6}$ and injected the output as an additive current to the current output of all controlled current sources. In order to inject noise into the voltage-dependent resistors, we used the white noise block and a scaling factor of 50 and injected the output as an additive component to the setting of the resistance value. Additionally, we limited the minimum value of the resistances to $0\,\varOmega$ .
